# Position parameters optimization of surface piercing propeller by artificial neural network

**DOI:** 10.1038/s41598-024-52325-8

**Published:** 2024-01-27

**Authors:** Masoud Zarezadeh, Nowrouz Mohammad Nouri, Reza Madoliat

**Affiliations:** School of Mechanical Engineering, Iran University of Science and Technology, Tehran, 16846-13114 Iran

**Keywords:** Engineering, Mathematics and computing, Physics

## Abstract

Improving the performance of surface-piercing propellers is achieved by investigating the influential factors. In this study, Artificial Neural Network is used to identify nonlinear models for estimating various phenomena. Non-Dominated Sorting Genetic Algorithm II is considered as an optimization tool. In this study, in order to optimize the position parameters, including the immersion ratio, angle of attack, and yaw angle, data from experimental tests at the HYDROTECH center of IUST were collected as the initial data field for the generation of training data by the artificial neural network, then experimental tests were implemented in the position of the Non-Dominated Sorting Genetic Algorithm II proposed as the output, and the results were compared. The Artificial Neural Network results showed that the mean error of the trained verified and test data is 7.5e−5, 1e−4, and 1e−4, respectively. Comparing the experimental and optimization results, the thrust coefficient showed a relative error of 9.7%, while the torque coefficient showed a relative error of 7.5%, this algorithm can be used as a cost-effective, time-saving method for a similar problem.

## Introduction

Surface-piercing propellers (SPPs) have been used specifically for high-speed racing boats and watercraft. SPPs eliminate the cavitation effect by applying the concept of supercavitation to the suction surface of the blades^1^. To overcome this challenge, the designers of high-speed racing boats have changed the installation position of the propeller. They aligned the shaft line with the waterline of the boat. This arrangement allows the propeller blades to rotate at the boundary between the water and air phases, creating an air entrapment effect that effectively reduces cavitation. Unlike conventional propellers, only part of the propeller comes into direct contact with the water, significantly reducing drag between the system components^2^. In addition, the complex physics and multiphase flow around SPPs have challenged researchers to gain a comprehensive understanding of how various parameters affect propeller performance. In 2007, Ferrando developed a standardized approach to designing propeller geometries that achieve the desired performance which has proven to be difficult^3^.

The available information on surface-piercing propellers is limited to a few specific geometries and is not easily accessible due to the relatively recent development and design of SPPs^4^. As a result, the design of such propellers has often been an iterative process or relied on the results of previous experimental studies conducted to data. In 2000, Dyson conducted various experimental studies to determine the factors influencing the design and performance of SPPs. These studies can be categorized into two groups: Research in the field of SPPs can be broadly divided into two main categories^5^. The first category focuses on identifying the key factors during the empirical testing phase, while the second category examines the nature and extent of the influence of the various factors on the proficiency of the propeller. In general, experimental methods are used to obtain hydrodynamic data on propeller performance, with scaled-down models being tested in water tunnels. Due to financial constraints, it is uncommon to perform tests on full-scale propellers. Shiba published the first experimental test results aimed at determining the influential parameters for hydrofoils and ventilation. Shiba proposed to consider ventilation independently of the characteristics of the surrounding air, such as pressure, and introduced dimensionless numbers that influence the performance of the propeller^6^. The results show that in cases where the Weber number exceeds 180, the behavior of the propeller aeration and the critical advance coefficient is independent of the effects of surface tension. In 1968, Hadler and colleagues carried out a study in which they examined several 3-blade surfaces-piercing propellers with the same surface conditions. During their investigation, they identified both total and partial aeration regions. Interestingly, in the partial aeration region, they observed the formation of air bubbles at the trailing edge of the blade^7^. This phenomenon led to an increased lift to drag ratio at this particular edge, resulting in improved efficiency of the propeller in this particular region. With total ventilation, the back of the blade is completely covered with air bubbles, which significantly reduces the efficiency of the SPP. In a study conducted by Shields, several supercavitation propellers were tested at different immersion depths. The results showed that the hydrodynamic coefficients exhibited independent behavior at Froude numbers above 4^8^. However, when Froude numbers were lower, an increase in the force exerted on the blade was observed. In a study by SHAO-ZONG in 1988, the researchers investigated the behavior of hydrofoils with different cross-sections when entering the free water surface^9^. Their results were consistent with the finding of Hadler and Hecker and Crown^7,10^, and showed similarities in the observed phenomena. Olofsson carried out a thorough investigation of the hydrodynamic properties of a 4-bladed propeller (841b)^11^. As part of the study, the forces and torques on a blade were measured using a transducer mounted in the hub to estimate the proficiency of the propeller. In addition, the influence of shaft angle and yaw angle on the efficiency of the propeller was considered. Nozawa and Takayama conducted a study to evaluate the performance of four different variants of 3-blade propellers. These propellers had different pitch configurations and were tested at different shaft angles and immersion depths^12^. To evaluate the stress on each blade during rotation, the researchers used multiple strain gauges on the blade surfaces. Through their analysis, they gained insights into the performance characteristics and structural integrity of the propellers under different operating conditions. In a study conducted by Ferrando et al.^13^, three surface-piercing propellers (SPP) were tested. The researchers found that the Weber number has a remarkable influence on the decisive forward coefficient and plays a crucial role in determining the force and torque coefficients within the entire ventilation surface. Their results highlighted the influence of Weber number on the performance characteristics of the propellers in terms of efficiency and propulsion. In 2007, Ding carried out an experimental test with several 6-blade propellers with different pitch ratios. The study compared the test results for three different Froude numbers between 3.46 and 4.24. The results showed that the behavior of the propellers at Froude numbers greater than 3.5 remained unaffected by both cavitation and Froude numbers indicating^14^. That the performance and characteristics of the propeller remained constant within the tested range despite the variations in these parameters. In 2010, Lorio conducted a study to investigate the effects of immersion depths, shaft angles and the yaw angle of the 4-blade propeller on performance. The experiments took place in a water tunnel facility. He found that both shaft angles had a significant influence on the performance of the propeller, indicating that they effectively influence the behavior and characteristics of the propeller^15^. In 2011, Florian used a genetic optimization algorithm to achieve several objectives, focusing on maximizing efficiency and reducing pressure fluctuations caused by the propeller^16^. The researchers also took into account the influence of the ship's propeller on the patterns near the stern of the ship. They used an iterative process to update the effective wake by using a segmented approach to model the flow around the hull. The results showed that the optimized propeller design successfully minimized pressure fluctuations and the occurrence of cavitation while maintaining the efficiency of the original design. Misra et al. studied the behavior of four different types of four-bladed propellers that had different blade section shapes and investigated the influence of Weber number under different immersion ratios and the change in the decisive advance coefficient. They also investigated the influence of the cup in generating the propeller thrust^17^. Vesting et al., developed an algorithm to improve the performance of the propeller by optimizing its blade geometry. The use of a genetic algorithm enabled an automated and systematic investigation of the design possibilities. The results can help to improve the efficiency of propellers and meet specific performance requirements under different operating conditions^18^. Nouri et al. focused on the optimization of CRPs using a combination of RANS-based computational fluid dynamics (CFD), genetic algorithm and the Kriging method. The results demonstrate the effectiveness of the proposed algorithm in optimizing propellers and highlight its potential to improve propeller performance and efficiency^19^. Seyyedi et al. have proposed an algorithm that deals with the selection of surface piercing propellers, taking into account the limitations of regression relationships and the importance of accurate determination of hydrodynamic coefficients. They investigated the effects of the position parameters and created a database of experimental data from various tests^20^. Yousefi and Shafaghat focused on understanding the occurrence, expansion and progression of the aeration phenomenon using the finite volume technique and the VOF model to replicate the behavior of the free surface. The results show that for advanced coefficients of 0.76–0.94 and radius ratios greater than 0.5, the area where the air penetrates completely disappears and the entire surface is in constant contact with the water^21^. Tadros et al. developed a methodology to optimize propellers by combining a propeller design software tool with a nonlinear optimization algorithm. The results showed that optimizing the propeller based on fuel consumption can reduce fuel consumption by up to 5.2% when focusing only on the efficiency of the propeller^22^.

Recently several researchers investigated advances method in machine learning applications as intelligent method. Rajhi et al., developed an optimized artificial intelligence model for predicting the surface properties of carbon fiber substance. The model includes an adaptive network-based fuzzy inference system (ANFIS) optimized through a Gazelle Optimizer (GO)^23^. Abdulsalam used some hybrid methods in their work such as Neural Network–Particle Swarm Optimizer (PSO-ANN) and Hybrid Neural Network–Humpback Whale Optimizer (HWO-ANN)^24^. Elsheikh comprehensively investigated the applications of ML methods such as support vector machine, artificial neural network, Gaussian process regression, K-nearest neighbor and presented results^25^. This research represents a novelty in the application of machine learning used in this study.

According to the previous studies in this regard, the development prediction estimator or an intelligent optimization procedure to predict the optimal positioning of the experimental models to create optimal performance of SPP propeller and reduced experimental cost and time has not been developed. On the other hand, to study the effects of each parameter, various tests and investigations have been carried out. Therefore, there is a need to implement a methodology to predict the effects of parameters on performance with acceptable decisions. In the present study, a methodology is introduced by combining Artificial Neural Network (ANN) and Non-Dominated Sorting Genetic Algorithm II (NSGA II) to optimize position parameters, including submergence depth, angle of attack, and yaw angle of SPP, and the hydrodynamic behavior of four blades SPP with different cross-sectional geometry is investigated. The tests were carried out using the water tunnel and the test equipment of the (IUST). The aim was to investigate the influence of different position factors on the hydrodynamic forces acting on the propeller at 10 different feed coefficients. Finally, experimental tests were carried out with the optimized position parameters and the results obtained were compared with the optimization results. The aim of including the optimization methodology in this study was to minimize the high costs and time associated with conducting extensive experimental tests.

## Propeller effective parameters

The basic parameters of a propeller, i.e. the dimensionless coefficients for thrust, torque and efficiency, must be evaluated about the propulsion coefficient assuming a steady flow and unobstructed water flow. These coefficients can be defined based on the forces acting on the propeller, the thrust coefficient, the force and torque coefficients, and the overall efficiency as follows^26^:

1 $$J=\frac{{V}_{a}}{nD}$$

2 $${K}_{T}=\frac{T}{\rho {n}^{2}{D}^{4}}$$

3 $${K}_{Q}=\frac{Q}{\rho {n}^{2}{D}^{5}}$$

4 $$\eta =\frac{{K}_{T}}{{K}_{Q}}.\frac{J}{2\pi }$$

**Figure 1:**
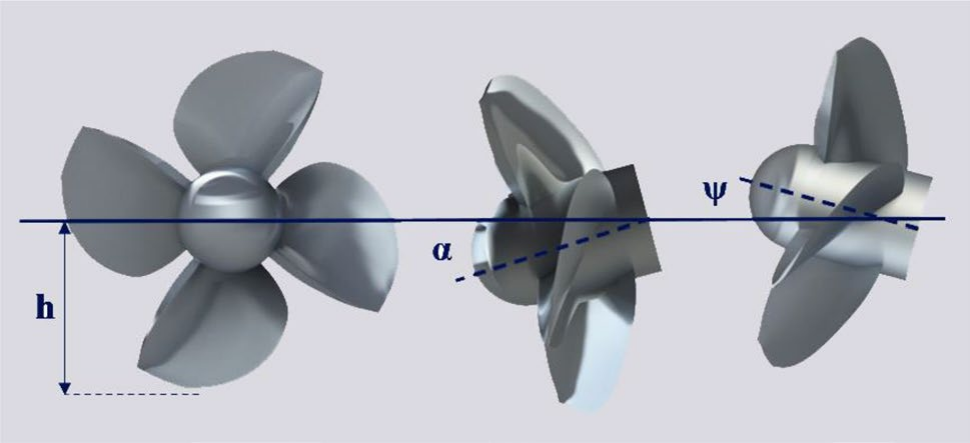
The positional characteristics of SPP to the free surface.

The hydrodynamic performance parameters of SPPs are influenced by various parameters that can be categorized into three main groups. The first group consists of position parameters that determine the position of propeller about the boundary between air and water and the direction of water flow. These parameters include the angle of attack (α), the yaw angle (ψ) and the immersion depth (${I}_{T}$)^26^. These parameters are shown visually in Fig. 1.

5 $${I}_{T}=\frac{{h}_{T}}{D}$$

The second category includes geometric parameters associated with the propeller's hub and blade configurations. These parameters consist of the propeller's diameter, pitch, number of blades, ratio of expanded area, and arrangement of the blade's pitch angle, tilt angle, and cross-sectional shape of the blade. These parameters define the physical dimensions and shape of the hub and blades of the propeller. The third classification includes parameters related to the operation and influenced by the characteristics and behavior of the flow. These parameters include the flow characteristics and speed of the water, the rotation speed and the atmospheric pressure of the propeller. These parameters are expressed by dimensionless numbers such as the Weber, Froude, Reynolds and cavitation numbers. These dimensionless numbers provide information about the flow conditions and help to evaluate the performance of the propeller under different operating conditions.

In general, the performance of the SPP can be assessed by analyzing its behavior as a function of the advance coefficient, which can be divided into three phases: the partial aeration surface at high advance coefficients, the transitional phase at medium advance coefficients, and the full aeration surface at low advance coefficients. Previous research has emphasized the importance of the Reynolds number in all three phases, and specific equations have been proposed to obtain its independence range. The Russian research institute KSRI (abbreviated name)^2^, has defined this independence range for the Reynolds number, as shown in Table 1. The Weber number represents the influence of the surface tension forces at the water–air interface and primarily influences the transition region and the critical advance coefficient and according to KSRI, the transition region and the critical forward speed coefficient. If ${W}_{n}\ge 180$, the coefficient (Jcr) remains unaffected by changes in the Weber number. The Froude number is a decisive factor that influences the performance of the propeller. Numerous studies have focused on evaluating its impact on the performance of SPPs. Since the propeller operates at the boundary between water and air, the Froude number is affected, which in turn affects the geometry of the propeller and the ventilation area. According to Table 1, the KSRI Institute has proposed $F{r}_{n}>3.5$ to maintain its independence.

**Table 1:** Equations and ranges of non-dimensional numbers effective on SPP performancey^27^.

Dimensional number	Equation and range	
Reynolds	${\mathit{Re}}_{n}=\frac{5n{D}^{2}({A}_{E}/{A}_{O})}{\nu Z}\ge 5\times 1{0}^{5}$	(7)
Weber	${W}_{n}=\sqrt{\frac{\rho {n}^{2}{D}^{3}}{\sigma }}\ge 180$	(8)
Froud	$F{r}_{n}=n\sqrt{\frac{D}{g}} \ge 3.5$	(9)
Cavity	$\sigma =\frac{{P}_{0}-{P}_{\vartheta }}{\frac{1}{2}\rho {{n}^{2}D}^{2}}$	(10)

The surface tension coefficient (σ), advance velocity (V), surface area ratio (AE/AO), cinematic viscosity (ν), propeller rotation (n), and blade number (Z) are parameters of equations.

Previous studies have recognized cavitation number as an important factor influencing the performance of SPPs. Similar to the vapor cavitation number, the cavitation number for SPPs is determined using Eq. (6), which is related to the advance velocity and atmospheric pressure^14^.

6 $${\sigma }_{0}=\frac{{p}_{0}-{p}_{\vartheta }}{\frac{1}{2}\rho {V}^{2}}$$

To maintain consistency between model tests and real-scale scenarios, it is essential to regulate the pressure of the surface of the water. Additionally, numerous studies have highlighted a correlation between the cavitation number and the Froude number. According to data from the KSRI Institute, when the cavitation number exceeds 1, the propeller's behavior in the specified operational zone remains unaffected by the cavitation number.

## Water Tunnel test

**Figure 2:**
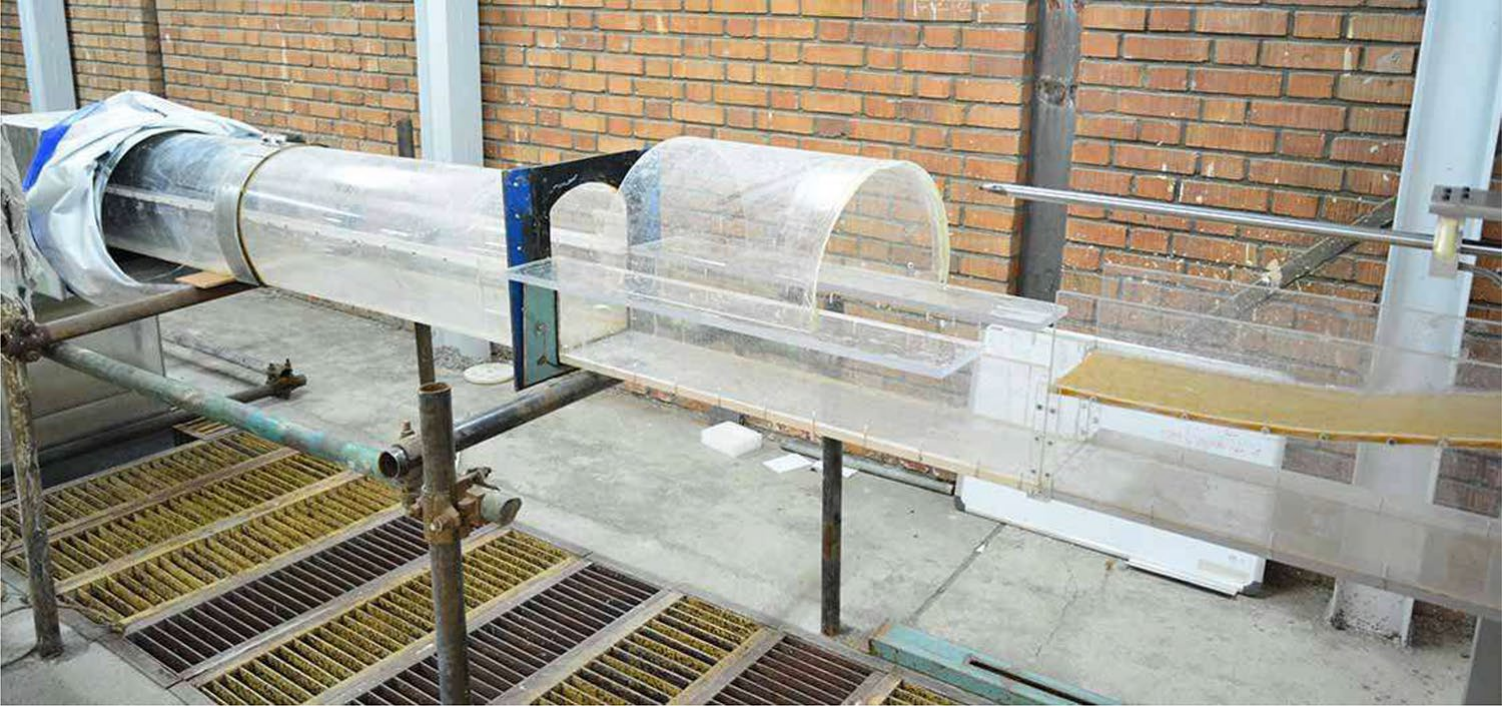
The water tunnel test of IUST.

Water tunnels serve as essential tools for measuring hydrodynamic forces acting on objects immersed or floating in water, as well as studying the fluid dynamics surrounding them. The water tunnel facility at IUST was purpose-built with specific features to facilitate fluid dynamics research in various areas, ensuring optimal testing conditions. The configuration of the tunnel, as shown in Fig. 2, consisted of an open section with dimensions of 25 × 20 mm. It offered a range of water velocities between 2 and 8 m/s. The tunnel system comprised basins, (1) five water pumps with (2) a combined flow rate of 1250 ${m}^{3}/h$, (3) a transfer pipeline, (4) flow control mechanisms, (5) an open test section, and (6) an open channel for water recirculation back to the basins. The basins served as the water supply source for the tunnel.

The conveying conduit featured a diversion valve for regulating the water flow velocity, along with a flowmeter for precise measurement and recording of the current flow rate. The test section, operating at atmospheric pressure, spanned a length of 1.5 m. This section was specifically designed to accommodate the propeller for testing purposes. The use of Plexiglas walls in this section enabled photography of the propeller and its interaction with the water flow.

The third module with a four-component dynamometer was used to measure the loads exerted on the SPP with the model's coordinate device and to align them with the propeller shaft. The dynamometer included force balances with two components to measure lateral forces and lift, a balance with one component to measure the thrust force on the propeller, and an S-load cell to measure the reaction torque of the propeller. Before conducting tests on the SPP propeller, the force-torque dynamometer underwent calibration to ensure accurate and reliable performance of the test mechanism. The calibration process followed the BBD (Box-Behnken Design) method and employed a multi-variable regression approach with a precision level of 95percent. The regression precision results are summarized in Table 2.

**Table 2:** The precision of the regression equation was determined for each channel of the force-torque dynamometer.

Source	Response			
Contribution	Lift (%)	Side (%)	Thrust (%)	Torque (%)
Model	99.99	99.91	99.92	99.99
Linear	99.98	99.44	99.90	99.99
Interaction	0.01	0.47	0.03	–
Error	0.01	0.09	0.08	0.01
Lack-of-fit	0.001	0.05	0.05	0.00
Pure error	0.01	0.04	0.03	0.01
R-predict	99.97	99.85	99.88	99.98

To establish the relationship between forces and the signals generated by the sensors, a 6-release calibration system was utilized at the IUST. Through this system, the hydrodynamic forces and torques acting on the model propeller in the water tunnel were measured and calibrated using an appropriate calibration model. This calibration step ensured that the recorded forces and torques accurately represented the hydrodynamic effects on the propeller during subsequent testing.

**Figure 3:**
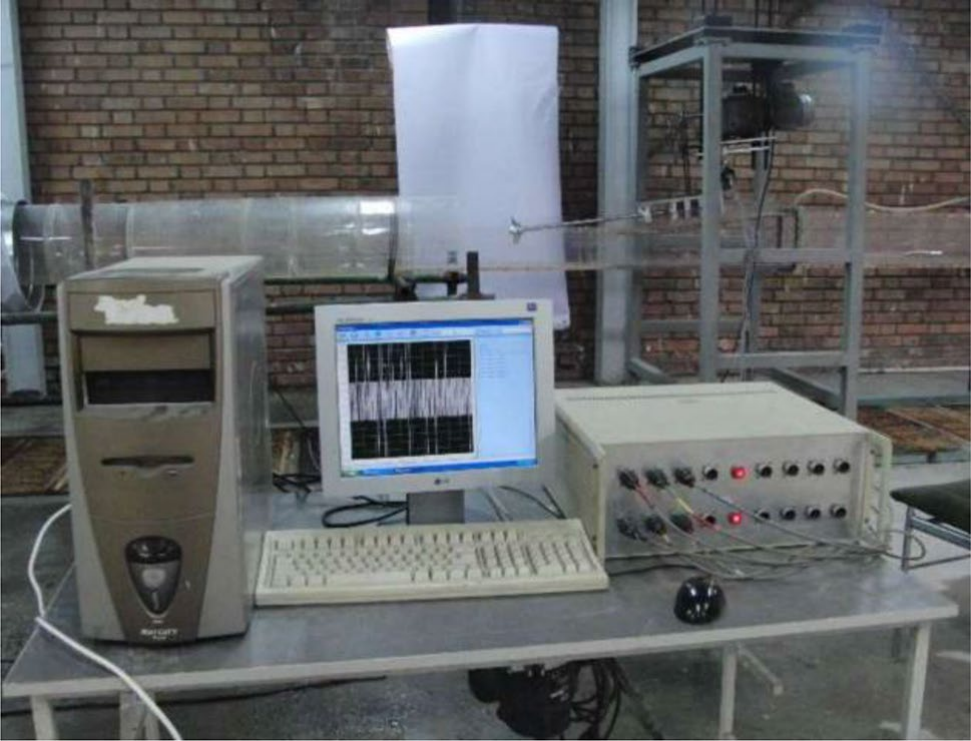
The data acquisition system.

The recorded data from the sensors was captured using a 16-channel data acquisition system (shown in Fig. 3). This system included signal conditioners, amplifiers, A/D signal converters, and specialized software. It could record data at frequencies ranging from 1 to 50 kHz. In this study, the system recorded the sensor signals at a frequency of 10 kHz with a time interval of 10 s. Subsequently, the recorded data underwent filtering and time-averaging processes.

## Model propeller

**Figure 4:**
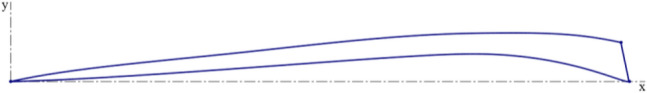
The section of SPP1 propeller.

**Figure 5:**
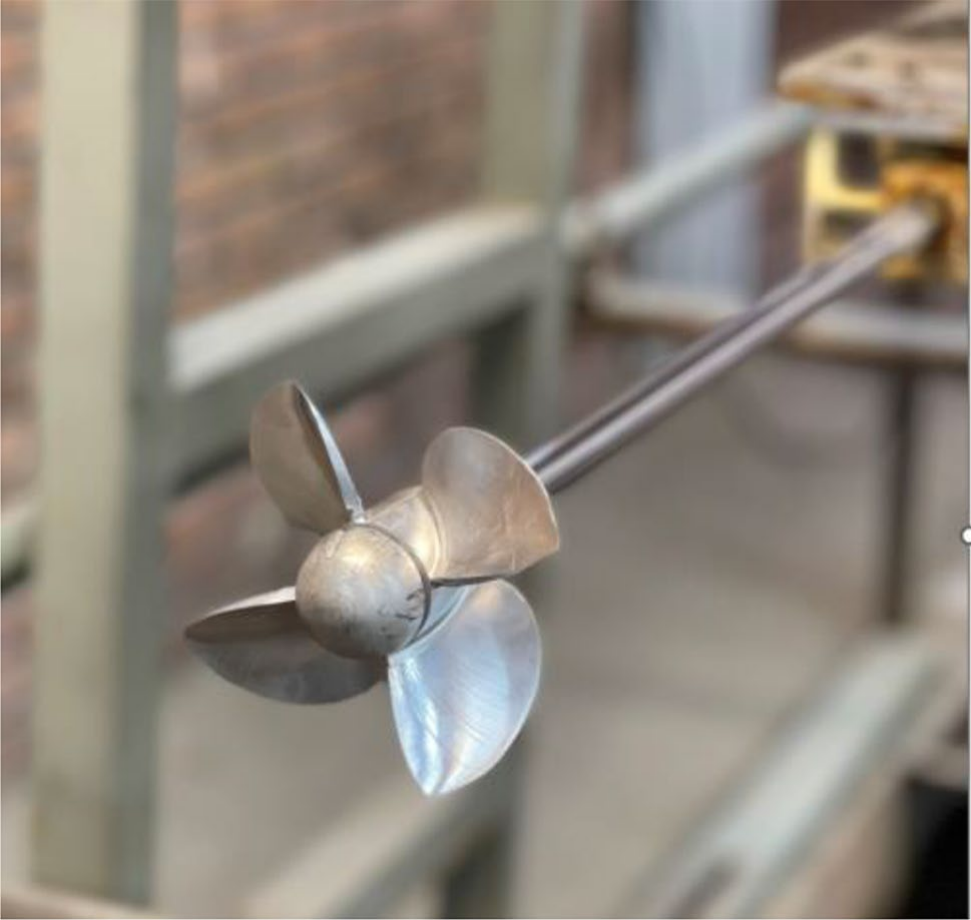
Propeller model (Spp1).

In the present study, a 4-blade SPP propeller with a section shown in Fig. 4 was used, which is referred to as SPP1 and was specially developed at the IUST Hydrotech Center. The geometrical details of the SPP specimen are depicted in Table 3. The visual representation of the constructed sample propeller is shown in Fig. 5.

**Table 3:** Specifications of SPP1 propeller.

Diameter model (m)	0.13	Count of propeller blades	4
Hub-diameter ratio	0.3	Turning direction	L.H
Expanded area ratio	0.58	Chord length (0.7R)	0.422
Material	AL	Pitch ratio	1.24

## Experimental test conditions for SPP1

To gain a comprehensive understanding of a propeller's characteristics, it is important to analyze its performance curve. Designers need this information to accurately estimate the initial thrust and efficiency when operating with small feed coefficients and to determine the interval in which thrust or maximum efficiency can be achieved. In addition, the performance of the propeller in the transient range is crucial due to possible vibration problems. In this study, the range of 0.4 ≤ j ≤ 1.4 was chosen based on the available capacity of the test setup. The tests prioritized the maximum velocity for each j performed under atmospheric conditions without controlling the cavitation number. In the tests, priority was given to the maximum velocity for each j, the experiments were conducted under normal atmospheric conditions without controlling the cavitation number, the value of ${F}_{n}$ was consistently maintained above 3.5 to minimize the influence of the cavitation number.

Table 4 contains further details. In addition, the influence of three different yaw angles on the water flow surrounding the propeller was investigated, whereby a fixed angle of attack of 6° was maintained. The specifications of the propeller included a diameter of 13 cm, $F{r}_{n}\ge 3.65$, a rotation range of 2000–3800 rpm, and an advance velocity of 3–6 m/s. These conditions ensured that ${\mathit{Re}}_{n}\ge 5\times 1{0}^{5}$ and considered the Weber number criteria based on velocity and rotation.

**Table 4:** Model propeller experimental test matrix.

Test factor	Variable factor		
	$I$	α	ψ
$I$	0.32,0.43,0.6	6	0
$\alpha$	0.32,0.43,0.6	3,6.5,8	0
$\psi$	0.4,0.6,0.5	6.5	4,7,10

Tests in physical conditions with Froude number 4 and Weber number above 200 had been done, and flow speed and rotation speed of the SPP in each advance coefficient has been considered based on the cavitation number 4.5 and between the advance coefficient 0.4 < J < 1.2.

## Optimization process

**Figure 6:**
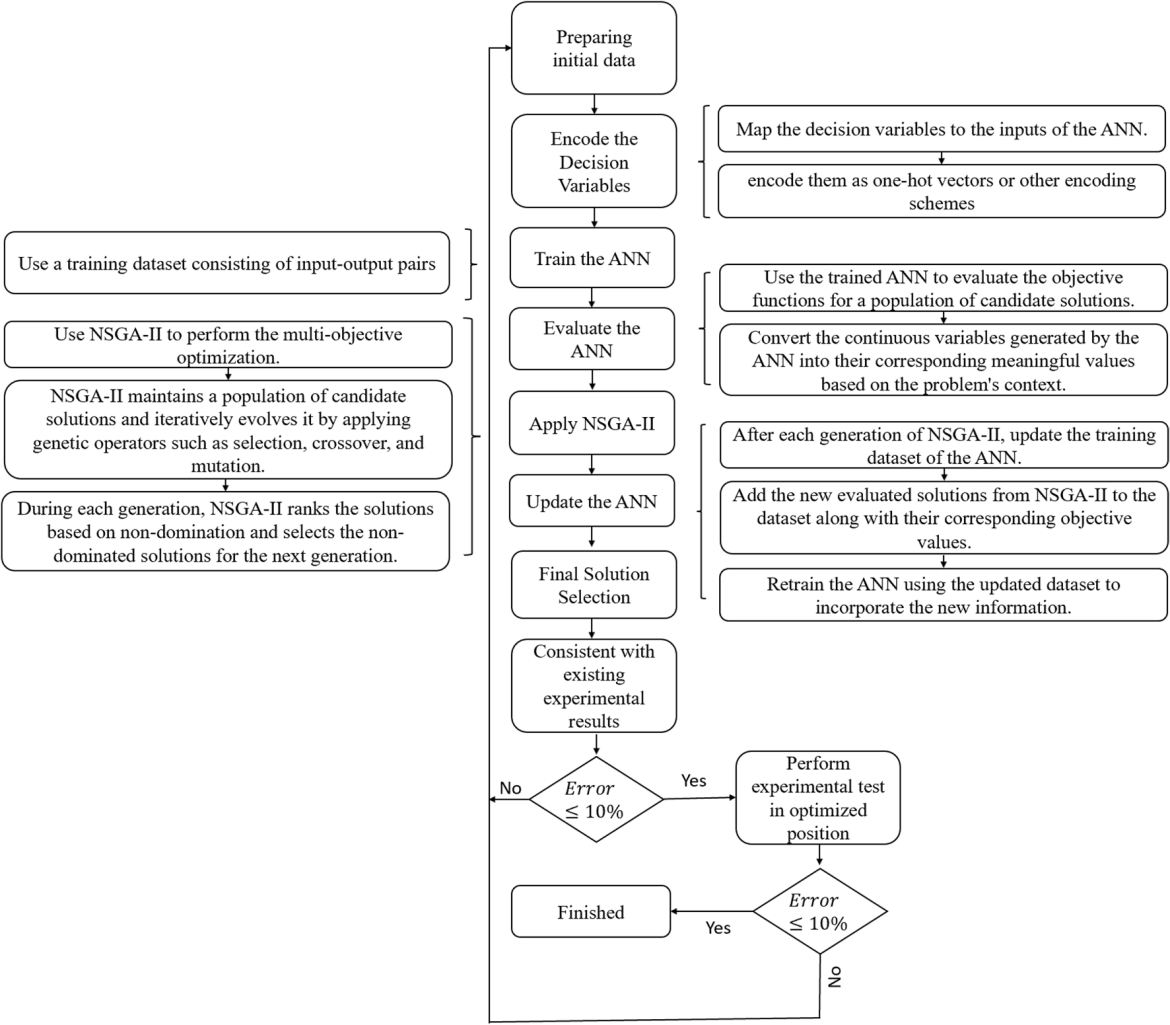
Optimization flowchart.

In this study, a combination of artificial neural networks (ANNs) and NSGA-II was used as an optimization method. Figure 6 shows this method.

In this study hydrodynamic parameters Kt and Kq are considered as objective function and immersion ratio, attack angle and yaw angle are variables. The initial population for the optimization process was derived from multiple experimental tests conducted on a 4-blade SPP1 model at the Hydrotech center of the IUST.

### Objective functions

The optimization problem had the following objective functions:

1. Maximizing the total thrust coefficient ($${f}_{1}=-{K}_{T})$$.
2. Minimizing the torque coefficient (${f}_{2}={K}_{Q}$)

the algorithm suggests a position where Kt is maximum and kq is minimum at the same time. This point is better for SPP than other points^28^.

### Artificial neural networks

Artificial Neural Networks (ANNs) have a history that can be attributed to the 1940s when^29^ introduced them in their work. They are credited with introducing the initial formal model of a neuron, which was inspired by the functioning of biological neurons. ANN is a computational framework that takes inspiration from the structure and operation of neural networks found in the human brain. It is a network of interconnected nodes, known as artificial neurons or nodes, which process and transmit information.

The formula of ANN involves the mathematical representation of the operations performed by individual neurons and the overall computation of the network^30^. Here are the key formulas involved in an ANN:

#### Neuron computation

The computation performed by a neuron involves taking the aggregate of weighted values of its inputs, applying an activation function, and producing an output. Let's consider a neuron with 'n' inputs, denoted as x_1_, x_2_, …, x_n_, and the associated coefficients w_1_, w_2_, …, w_n_. The bias term is represented by b. The weighted sum of inputs, also known as the linear combination, is calculated as:

$$ {\text{the sum of weighted values }} = \, \left( {{\text{w}}_{1} *{\text{x}}_{1} } \right) + \left( {{\text{w}}_{2} *{\text{x}}_{2} } \right) + \cdots + \, \left( {{\text{w}}_{{\text{n}}} *{\text{x}}_{{\text{n}}} } \right) + {\text{b}} $$

The linear combination is then passed through an activation function, denoted as f(), to incorporate non-linear transformations and generate the neuron's output:

$$ {\text{Neuron - output }} = {\text{ f}}\left( {\text{linear - combination}} \right) $$

#### Activation functions

Activation functions are utilized to introduce non-linear characteristics to the output of a neuron, enabling the neural network to capture and represent intricate relationships. Common activation functions include^31^:

$$ \begin{gathered} {\text{Sigmoid}}:{\text{ f}}\left( {\text{x}} \right) \, = { 1 }/ \, \left( {{1 } + {\text{ exp}}\left( { - {\text{x}}} \right)} \right) \hfill \\ {\text{Hyperbolic Tangent }}\left( {{\text{tanh}}} \right):{\text{ f}}\left( {\text{x}} \right) \, = \, \left( {{\text{exp}}\left( {\text{x}} \right) \, - {\text{ exp}}\left( { - {\text{x}}} \right)} \right)/\left( {{\text{exp}}\left( {\text{x}} \right) \, + {\text{ exp}}\left( { - {\text{x}}} \right)} \right) \hfill \\ {\text{Rectified Linear Unit }}\left( {{\text{ReLU}}} \right):{\text{ f}}\left( {\text{x}} \right) \, = {\text{ max }}\left( {0,{\text{ x}}} \right) \hfill \\ {\text{Softmax }}\left( {\text{used in the output layer for multi - class classification}} \right):{\text{ f}}\left( {\text{x}} \right) = {\text{exp}}\left( {\text{x}} \right)/{\text{sum}}\left( {{\text{exp}}\left( {\text{x}} \right)} \right) \hfill \\ \end{gathered} $$

#### Forward propagation

In the forward propagation step, the inputs of the network are transmitted through the various layers of neurons to produce the final output. The output of each neuron in a layer serves as the input received by the neurons in the subsequent layer. The computation involves performing the neuron computation formula for each neuron in the network, layer by layer until it reaches the output layer.

#### Loss function

The loss function evaluates the discrepancy between the predicted output of the neural network and the actual output, quantifying the level of discrepancy. The selection of the loss function is determined by the specific objective of the task, for instance, the mean squared error (MSE) is commonly utilized as a loss function to quantify the average squared deviation between the predicted and actual values, and categorical cross-entropy for classification tasks. The specific formula of the loss function varies based on the problem being solved.

#### Backpropagation

Backpropagation is used to update the weights of the neurons based on the error or loss computed during forward propagation. The gradient of the loss with the network's weights is calculated using the chain rule of calculus.

The updated weight value is obtained by multiplying the gradient with a learning rate and subtracting it from the current weight value.

(ANN) can be conceptualized as a loop that iteratively performs a sequence of steps to process input data and adjust its internal parameters. Here is a description of the ANN loop:

**Step 1**: Input Data.

The loop begins by receiving input data, which could be numerical features, images, text, or any other form of structured or unstructured data.

**Step 2**: Forward Propagation.

The input data is propagated via the network in a forward direction. Each neuron in the network receives weighted inputs, performs a computation, and produces an output signal based on an activation function. the output of one layer serves as the input for the next layer, enabling the flow of information throughout the network.

**Step 3**: Output Calculation.

The ANN generates an output based on the final layer's activation. This output could be a class label, a prediction, or a probability distribution, depending on the specific task the ANN is designed for.

**Step 4**: Error Calculation.

The output of the artificial neural network (ANN) is compared to the expected or desired output, and a measure of the error or discrepancy is calculated. This error metric quantifies the discrepancy between the predicted output and the expected output.

**Step 5**: Backpropagation.

The error is then propagated backward through the network using the backpropagation algorithm.

The algorithm calculates the contribution of each neuron and its associated weights to the overall error, allowing for adjustments to be made to the network's parameters.

**Step 6**: Parameter Update.

The parameters of the ANN, including the weights of the neurons, are modified based on the error calculated during backpropagation. Optimization algorithms, such as gradient descent or its variants, are commonly used to update the parameters in a way that reduces the error and improves the network's performance.

**Step 7**: Loop Iteration.

The loop iterates, repeating steps 2–6, with new input data or a batch of input data, to further refine the network's performance. The number of iterations or epochs can be predetermined or based on convergence criteria, such as reaching a certain level of error or stability in the network's performance. The loop continues until a stopping condition is met, such as a predefined limit on the number of iterations or the desired level of accuracy is achieved. The training loop enables the ANN to learn from the input data, adjust its parameters, and iteratively improve its predictions or outputs. Once trained, the ANN can be used for making predictions or classifications on new, unseen data.

It is significant to emphasize that the formula of an ANN is a generalized representation, and the specific implementation and variations can differ based on the architecture, activation functions, loss functions, and other factors chosen for a particular neural network^32^.

### NSGA II

NSGA-II, also known as Nondominated Sorting Genetic Algorithm II, is a popular optimization technique utilized for solving problems that involve multiple objectives that may conflict with each other. It is an extension of the original NSGA algorithm and is specifically designed to handle multiple objectives efficiently. the NSGA-II operates based on the principles of genetic algorithms, which draws inspiration from the principles of natural evolution to guide its optimization process. It maintains a population of candidate solutions, known as individuals, and iteratively evolves this population to generate better solutions^33^.

Here is a step-by-step description of how NSGA-II works:

Initialization:

- Create an initial population P0 of size N.
- Each individual in P0 is expressed as an array of decision parameters, denoted as X.

Fitness evaluation:

Evaluating the quality of each individual in P0 relied on the multiple objectives of the problem. Denote the fitness of individual i as F(i).

Non-dominated sorting:

- Perform non-dominated sorting to classify individuals into different fronts.
- Let Fi be the set of individuals that outperform individual i.
- Let S(i) be the set of individuals that are surpassed by individual i.
- Assign a non-domination rank R(i) to the individual i, indicating the front it belongs to.
- Calculate the dominance count N(i) for each individual i, representing the count of individuals that outperform i.

Crowding distance assignment:

- Calculate the crowding distance D(i) for each individual i in each front.
- The crowding distance measures how crowded an individual is in the objective space.
- For each objective function j, calculate the distance between the two neighboring individuals (Nbr1 and Nbr2) in the same front.
- Sum up the distances for all objectives and assign the result as the crowding distance for individual i.

Selection and reproduction:

- Select parents from the current population P based on non-dominated sorting and crowding distance.
- To favor individuals in less crowded regions, the selection process is based on a binary tournament.
- Randomly select two individuals and compare their non-domination ranks. Choose the individual with a better rank or, if the ranks are the same, the one with a larger crowding distance.
- Perform crossover and mutation operations on the elected parents to create offspring.
- Combine the offspring and parents to form the next generation P'.

Population update:

- Create an empty population Q.
- Apply non-dominated sorting and crowding distance calculation on P' to classify individuals into different fronts and calculate crowding distances.
- Until Q reaches the desired population size N, repeatedly select the front with the current rank R and add individuals to Q until the size limit is reached.

Termination:

- Continue the selection, reproduction, and population update steps for a predetermined number of generations or until a condition is satisfied.
- The termination criterion can be a maximum number of generations, a desired level of convergence, or the attainment of a satisfactory solution.

By following these steps, NSGA-II efficiently searches for a diverse set of solutions along the Pareto front, balancing exploration and exploitation to handle multi-objective optimization problems.

## Results and discussion

The ANN was trained in a supervised manner based on the experimental results of the SPP, whose specifications are collected, and the training stopping criteria include the mean square error, gradient error, number of periods, and number of verified periods. The stopping ranges are also listed in Table 5, accordingly, the training algorithm is stopped at 8.66e−08.

**Table 5:** Training progress of ANN.

Unit	Initial value	Stopped value	Target value
Epoch	0	181	300
Elapsed time	–	00:00:05	–
Performance	0.0188	1.14e−05	1e−06
Gradient	**0.00132**	**8.66e−08**	**1e−07**
Mu	0.001	1e-08	1e+10
Validation checks	0	149	300

Significant values are in bold.

**Figure 7:**
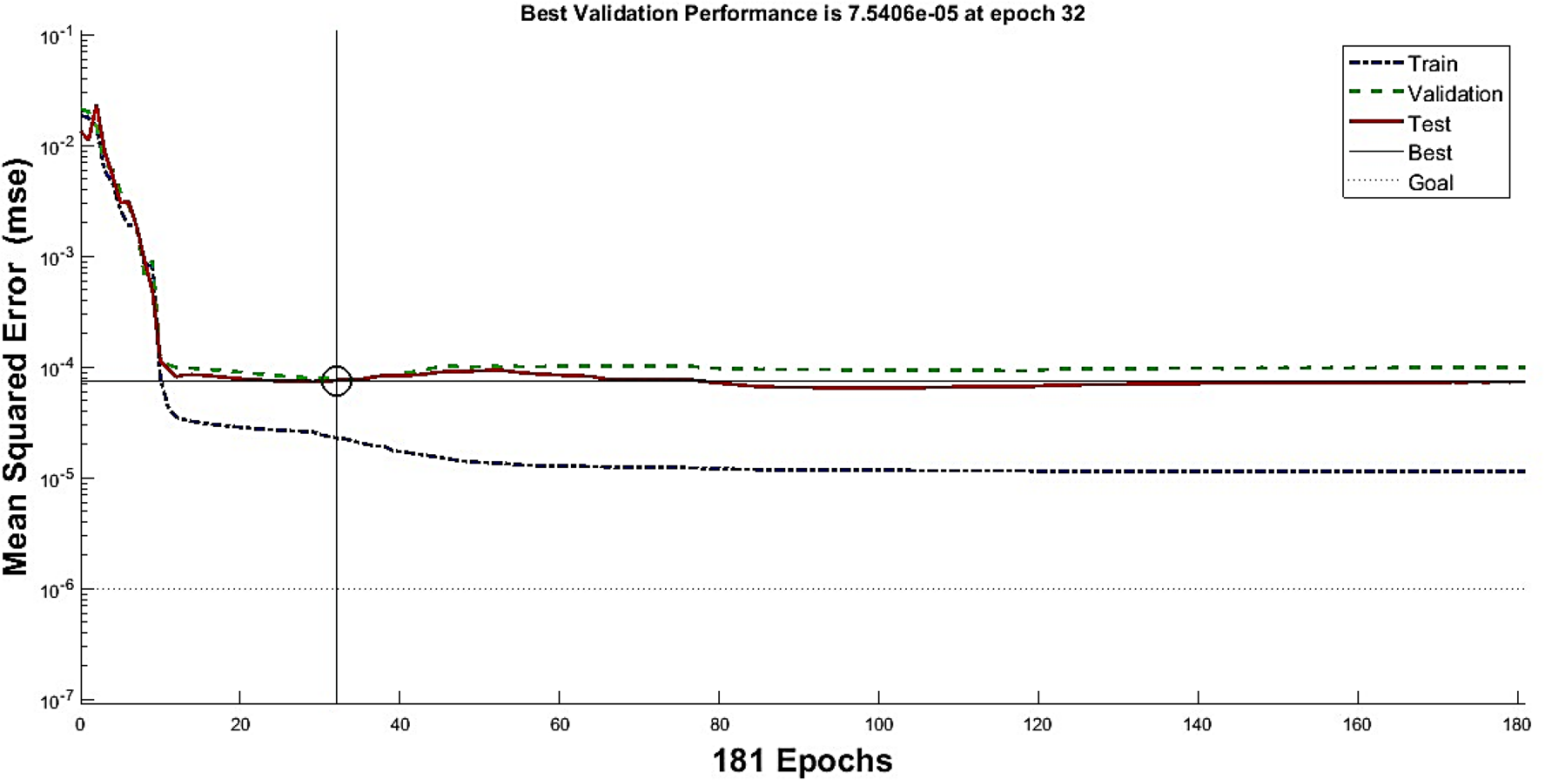
The average squared difference in training progress.

**Figure 8:**
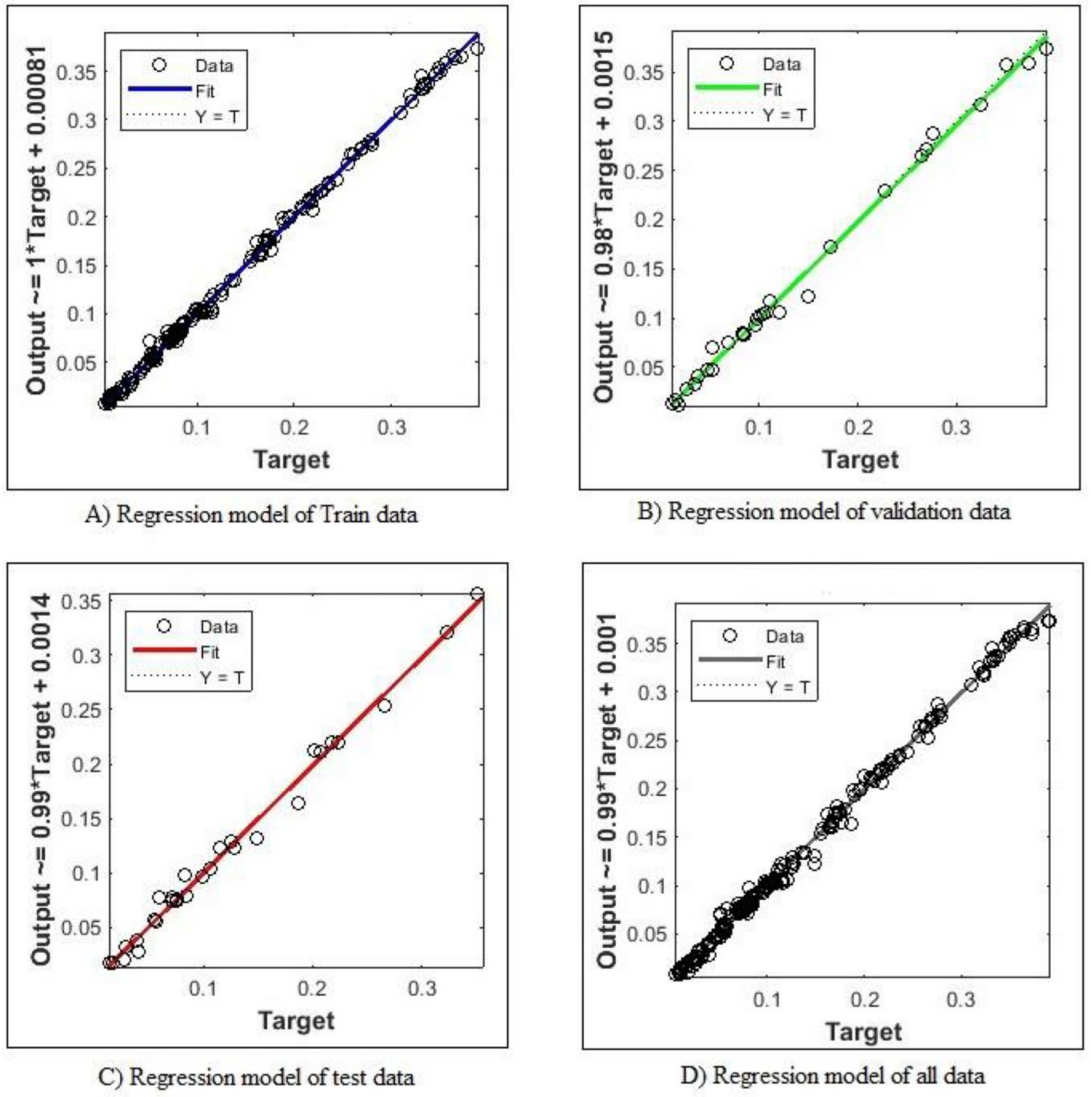
The regression model between target and output (**a**) train data (**b**) validation data (**c**) testing data (**d**) entire data.

Figure 7 also shows the changes in the mean square error of the training dataset, the verification and the test data for different time periods. As is specified, the algorithm finished the training in the 32nd epoch with a mean square error of 7.5e−5. The mean square difference of the test and verification data is also 1e−4. In this study, many models have been examined to find the best ANN algorithm for creating new data. Due to the non-uniqueness of the output of the trained algorithms, the researchers sought to find the best answer. The criterion chosen to validate the artificial intelligence algorithm used in this research is a linear regression model. The regression model can be examined for the training, verification, and test data to assess the precision of the ANN. Figure 8 shows the error between the target value and the results obtained. As can be seen in Fig. 8, the slope and intercept of the regression between the target value and the results obtained were almost 0.99 and 0.

According to Fig. 8, ANN can be used in the given range to estimate the properties of SPP propellers with different position parameters.

**Figure 9:**
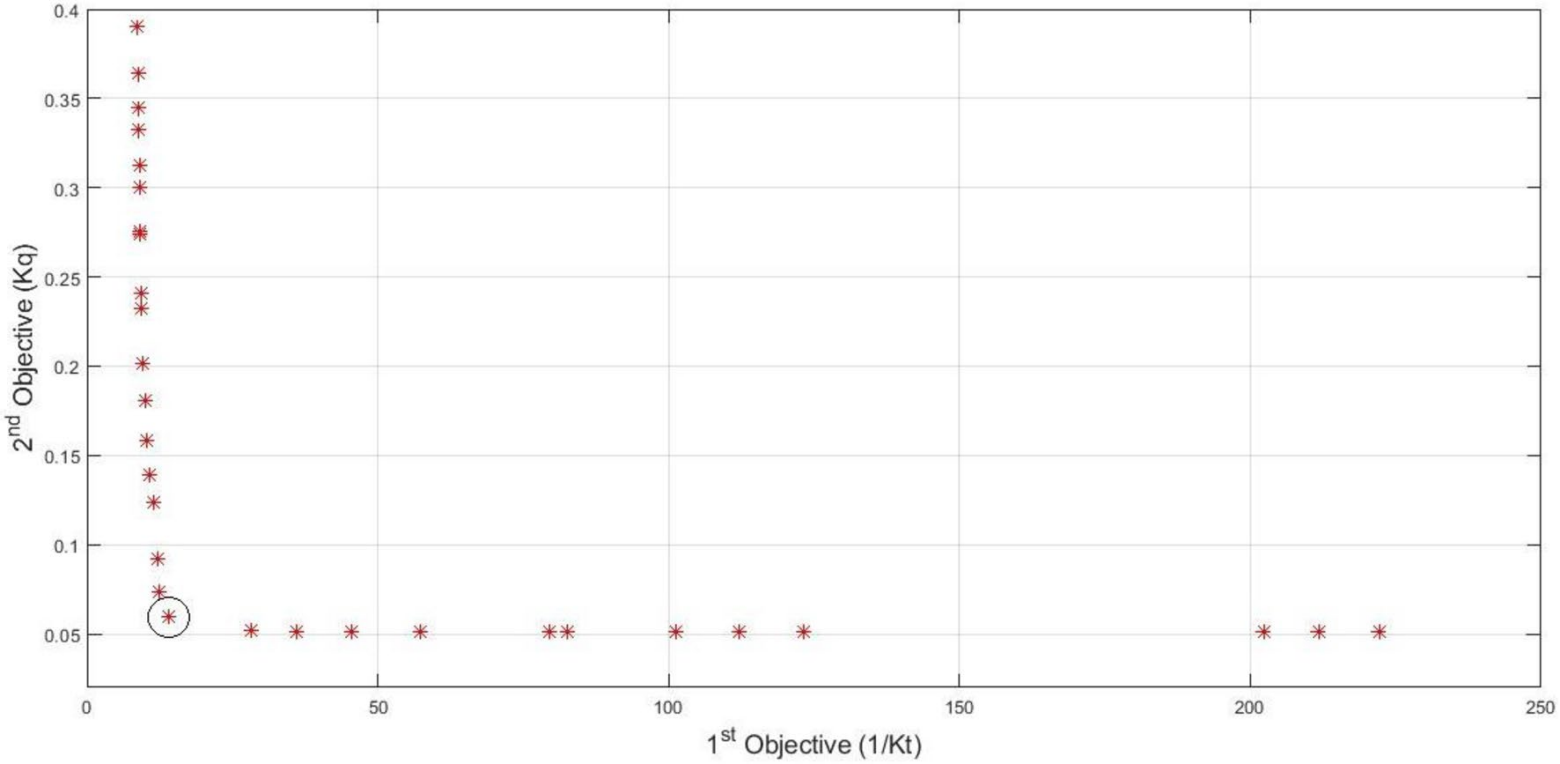
Pareto front of the best results.

After the desired algorithm has been trained, (NSGA II) is used to find the best answer. As mentioned before, in this study, an attempt is made to find the best position for the propeller based on the lowest torque coefficient and the highest thrust coefficient. Therefore, the first cost function was considered as the inverse of the thrust coefficient and the second cost function as the torque coefficient. Figure 9 shows the Pareto of the best solutions obtained using a genetic algorithm (NSGA II). The best solution is selected from the various points in such a way that the distance of the best solution from the zero coordinate has the smallest value. Based on the results obtained, the highest thrust coefficient is 0.0717, while the lowest torque coefficient is 0.0595. Based on this answer, the positions of alpha, Y, I and j are obtained as 3.4878, 5.1463, 0.4000 and 0.8890 respectively.

**Figure 10:**
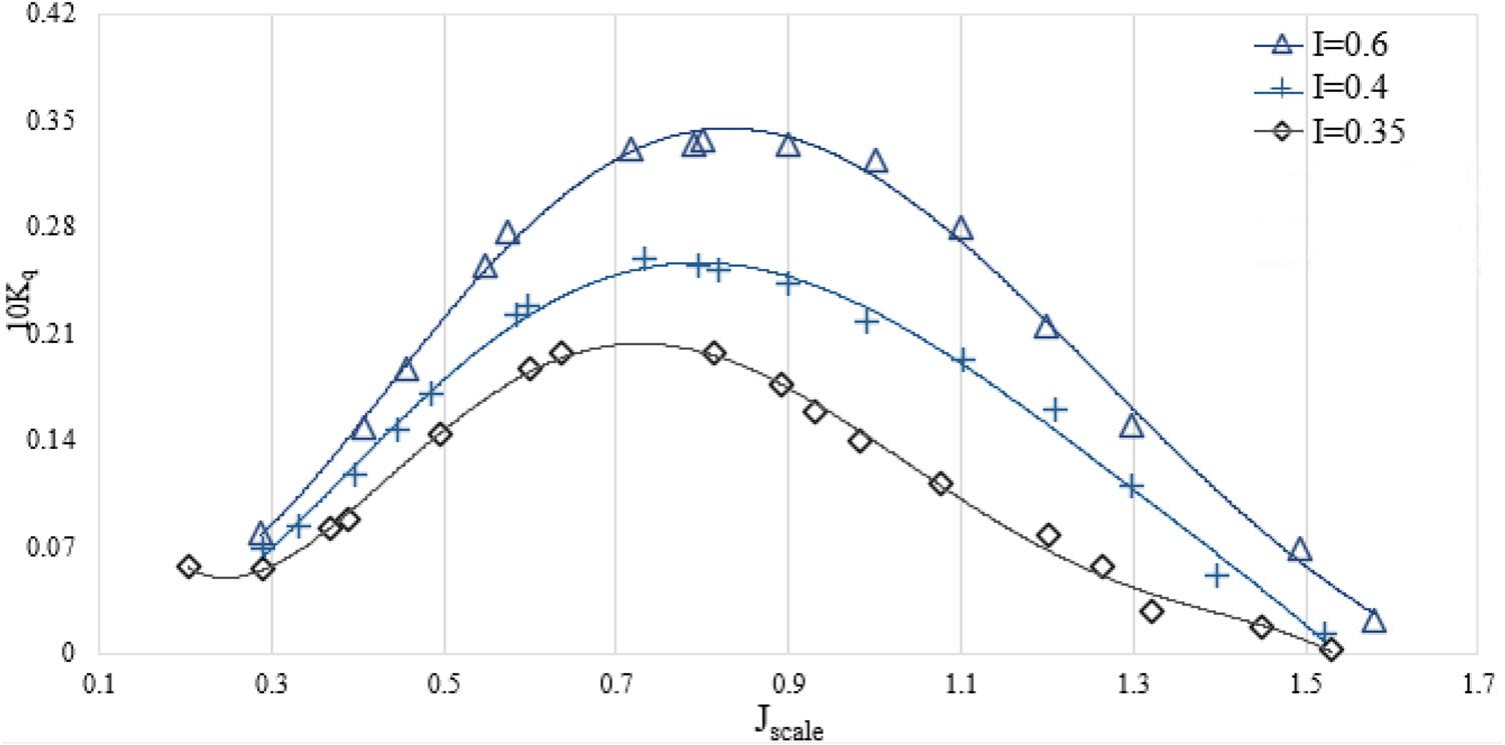
The influence of the submergence level on the torque factor in the orientation of SPP at an angle of 6°.

**Figure 11:**
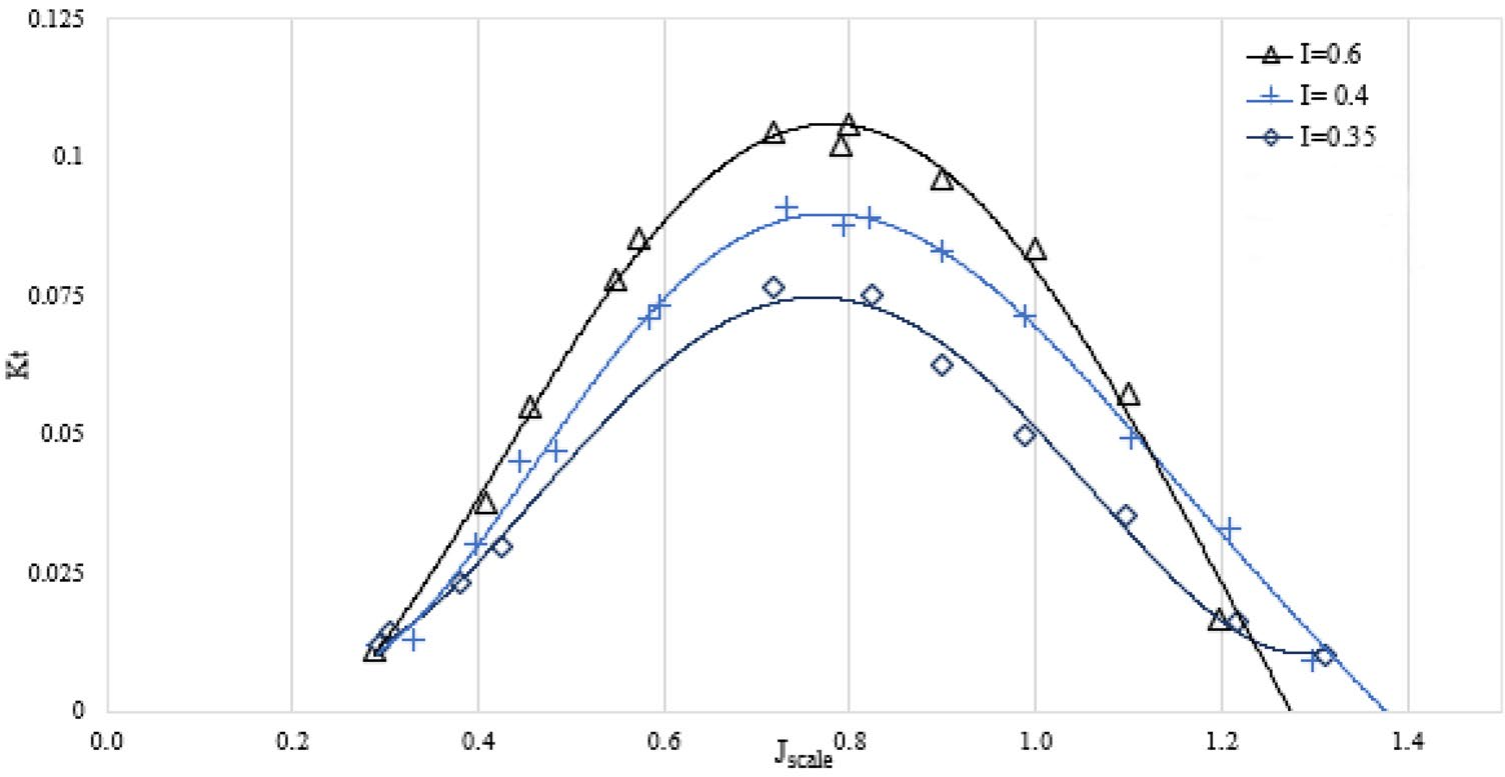
The relationship between the submergence level and the thrust factor in the orientation of SPP at an angle of 6 degrees.

Figures 10 and 11 show the relationship between the change in the immersion depth of the SPP and the corresponding torque and thrust coefficients. The results show that an increase in the immersion depth leads to an increase in the coefficients for torque and thrust. Reduced immersion depth will in reality have an effect on propeller effective disc place and evolved air flow hollow space at the back of the propeller. These parameters affect the drag and lift exerted at the propeller and have an effect on the torque and thrust at any advance ratios. In addition, decreasing the moist region of the SPP additionally reduces drag and may enhance propeller proficiency.

**Figure 12:**
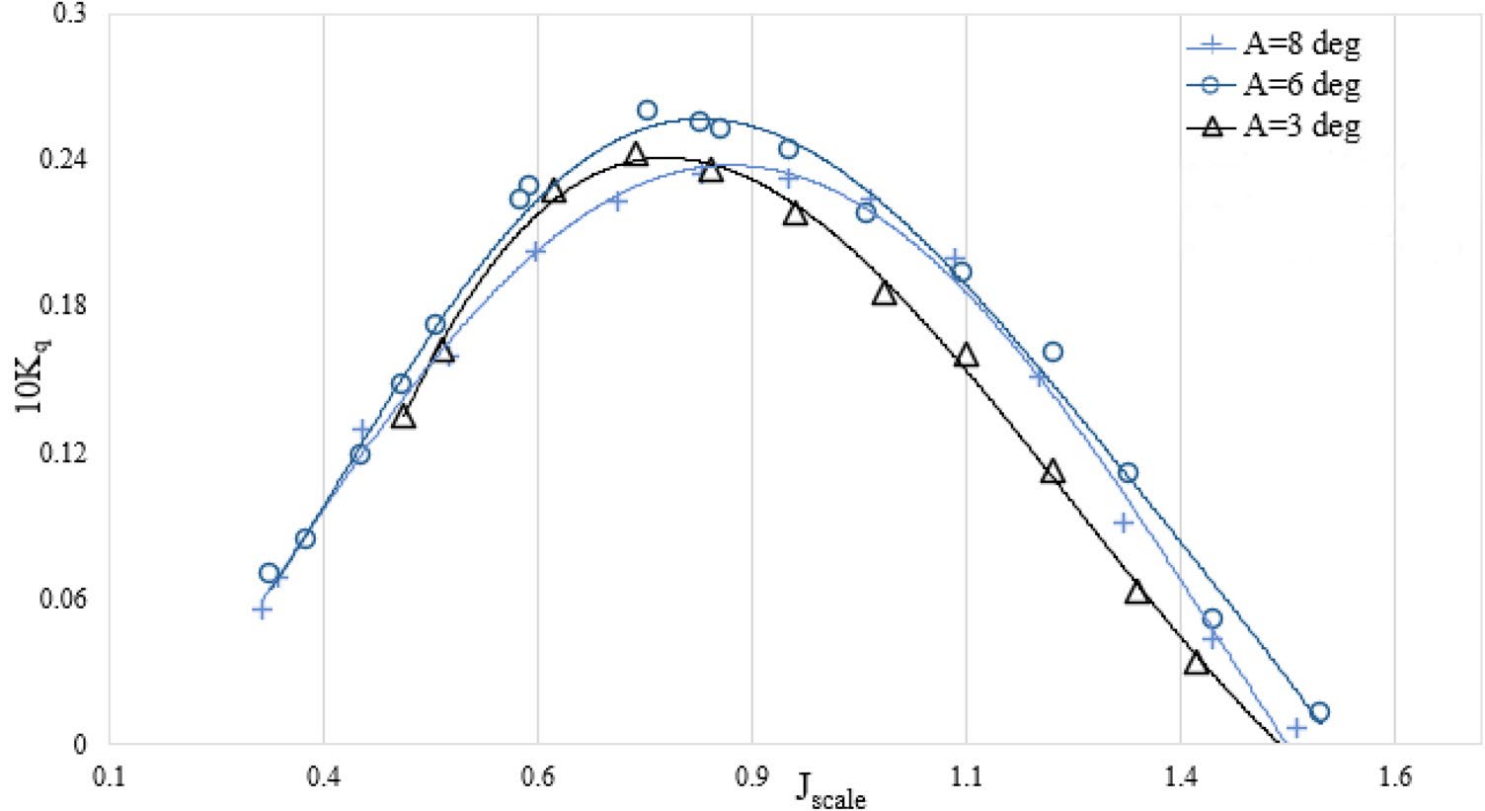
The torque factor on propulsion orientation in various attack angles (${I}_{T}$=0.4).

**Figure 13:**
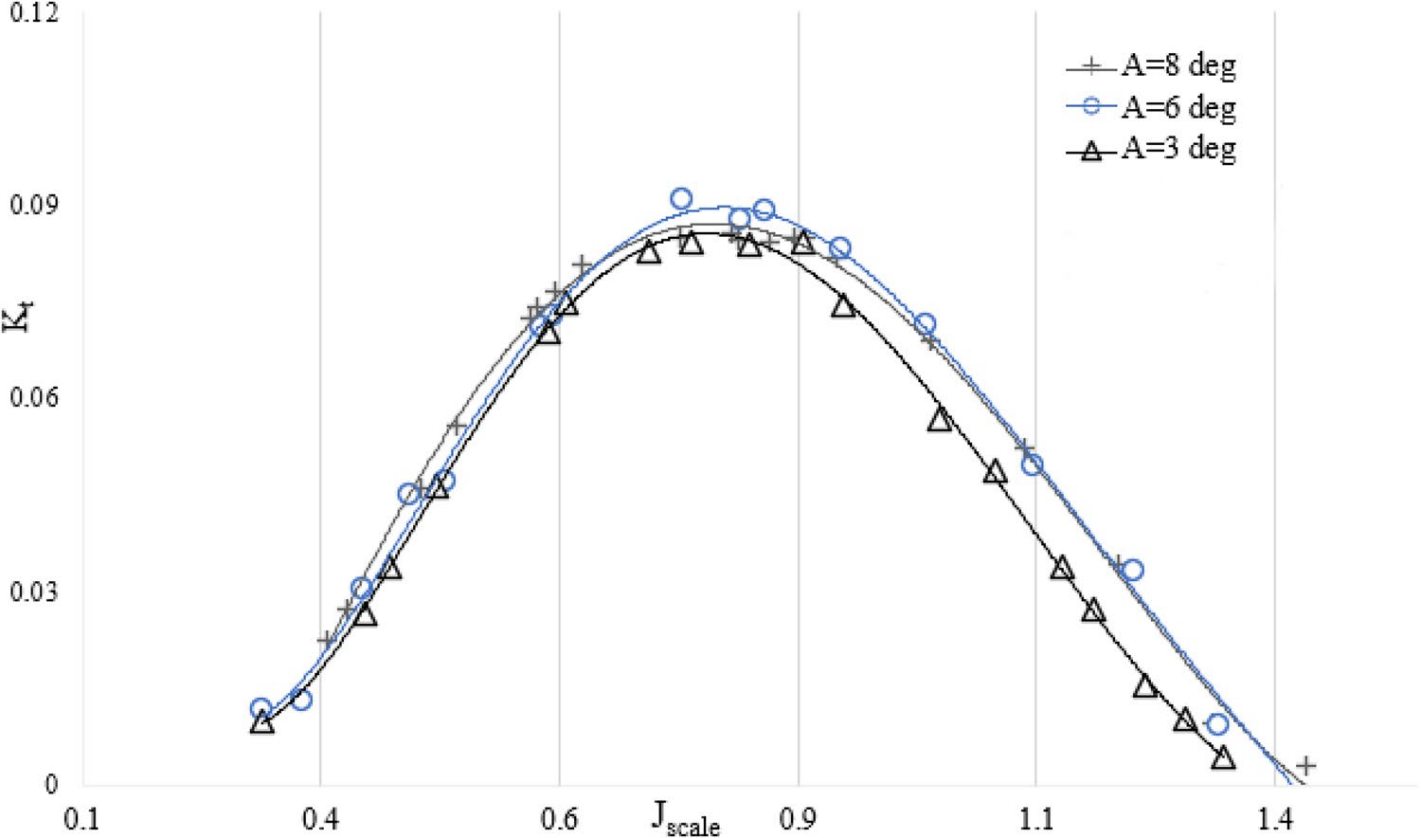
The thrust factor on propulsion orientation in various attack angles (${I}_{T}$=0.4).

Figures 12 and 13 show the relationship between the variations in the angle of attack of the SPP and the resulting coefficients for thrust and torque within the immersion range of 0.4. The results show that as the angle of attack increases, there is a remarkable improvement in both the torque and the axial thrust coefficient. Due to the asymmetric operating conditions and the vibrations of the SPP, significant horizontal and vertical forces are generated. Therefore, the route of the forces is anticipated to be with inside the route of propulsion through converting the angle of attack and growing the performance of propulsion. Therefore, an improvement in torque and thrust coefficients is observed.

**Figure 14:**
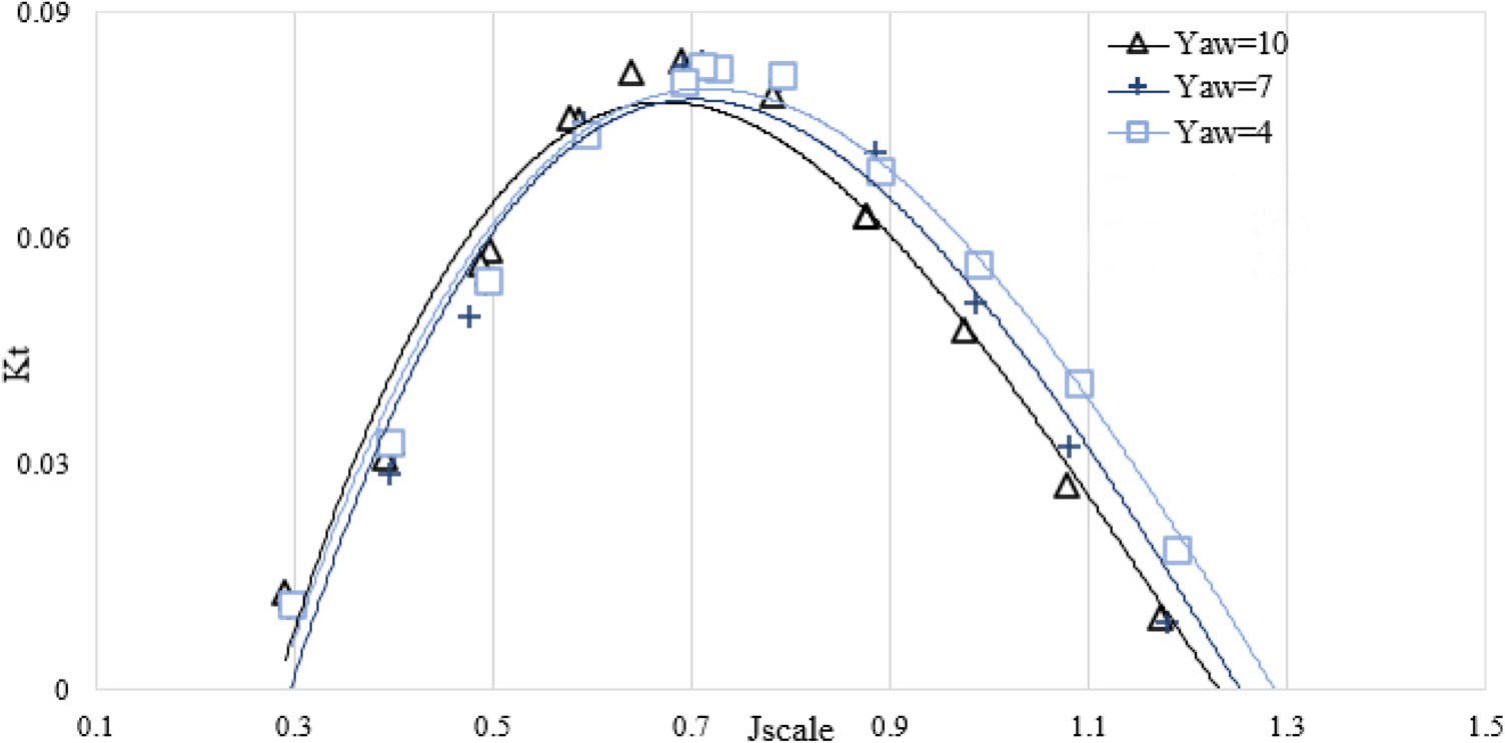
The relationship between the thrust factor and the yaw angle in the orientation of the propeller (${I}_{T}$=0.4).

Figure 14 shows the decrease of the axial thrust when the yaw angle increases. This is because the change in the vertical axis reduces the effective pitch of the propeller in the direction of the flowing water.

**Figure 15:**
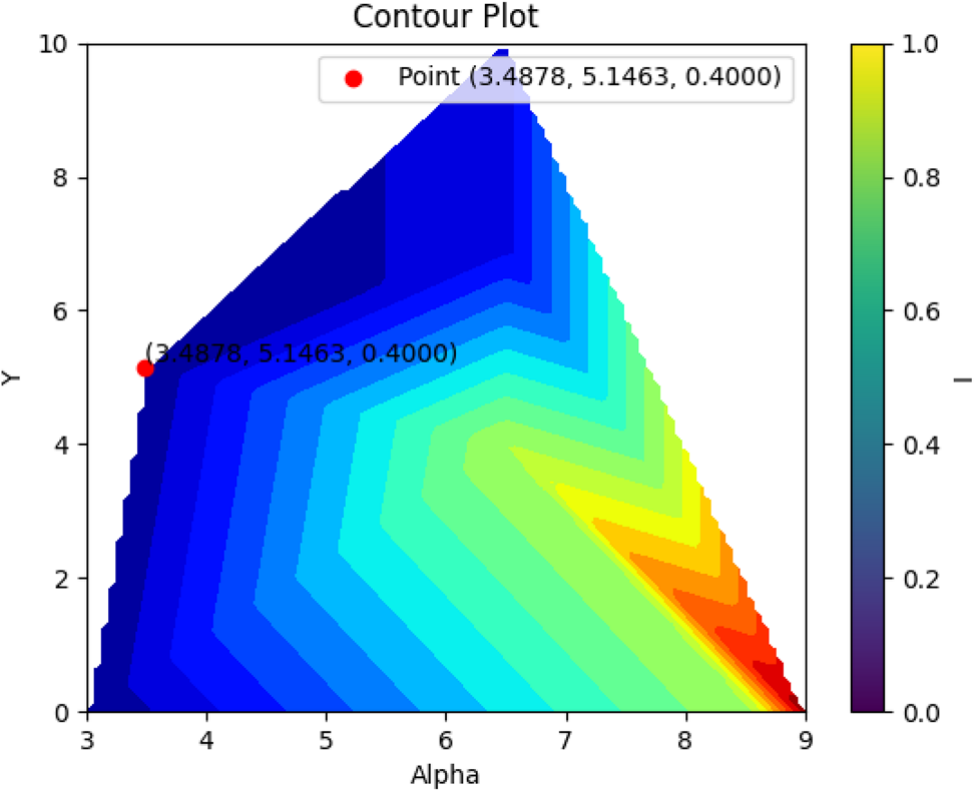
The contour plot represents the optimal attack angle, immersion depth, and yaw angle of the SPP.

**Figure 16:**
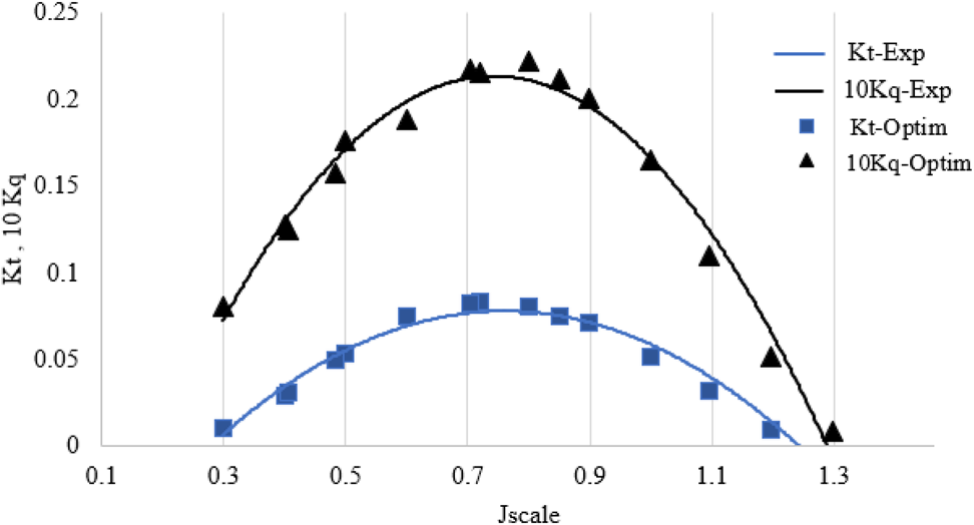
The comparison between the changes in hydrodynamic coefficients for the optimized position and experimental data.

**Figure 17:**
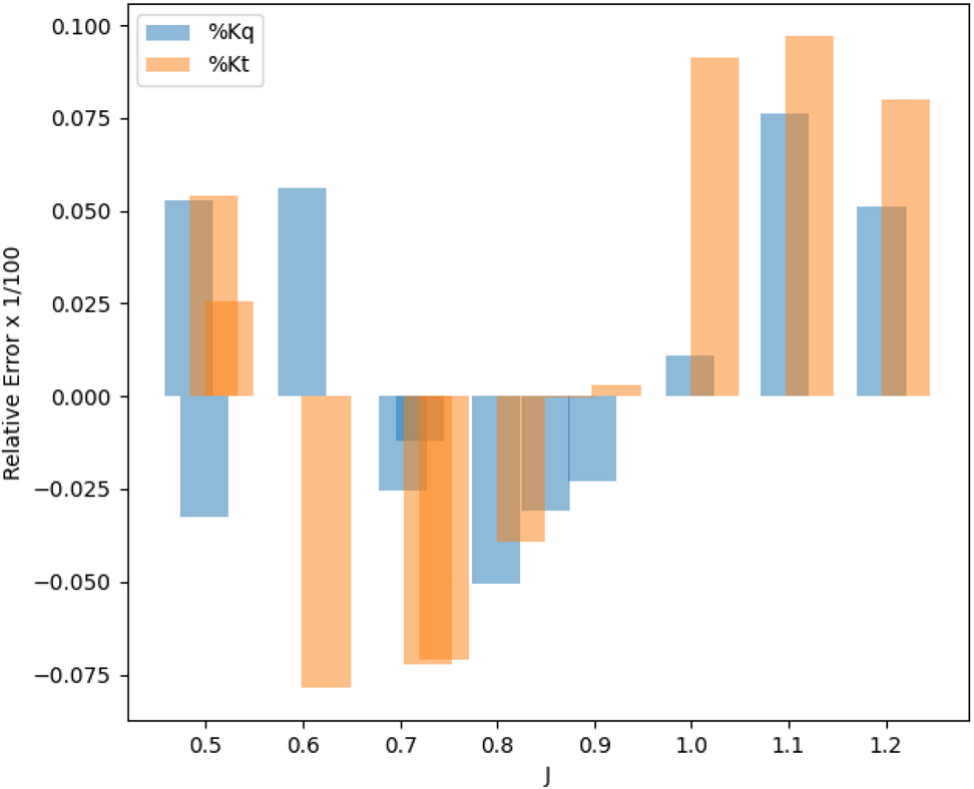
The relative error between optimization results and experimental outcomes in different J.

**Figure 18:**
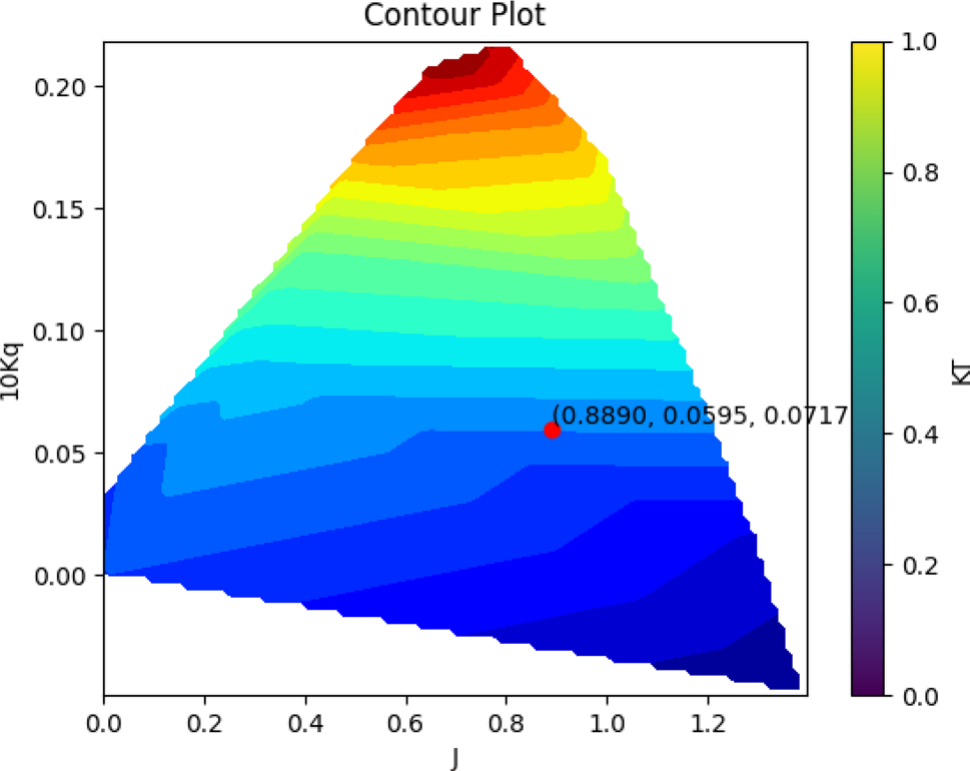
The hydrodynamic coefficients of the optimized point.

In Fig. 15, the optimal point resulting from the optimization process is highlighted as a red data point. This optimal point represents the best performance of the SPP based on experimental tests conducted at the IUST Hydrotech Center. The validation of this optimal point is confirmed in Fig. 18, where it can be seen that the maximum KT and minimum Kq values occur close to the j value of approximately 0.8890. Figure 16 demonstrates a comparison between the experimental test and the optimization results, and the relative errors based on J are shown in Fig. 17, with the maximum relative error for the thrust (9.7%) and torque (7.5%) factors at J = 1.1.

As shown in Fig. 18, the optimized output of the SPP corresponds to a KT value of 0.0717 and a Kq value of 0.0595. These values resulted in the best performance of the SPP through the optimization process.

## Conclusion

The aim of this study is to find the best parameters of SPP position using artificial intelligence. First, a data matrix is created using laboratory data. Then new data was created using ANN algorithm and the best position parameters were extracted using NSGA II optimization algorithm.

Preliminary data were collected from experimental tests in the IUST water tunnel, focusing on variations of the hydrodynamic coefficients with respect to the position parameters. The parameters considered were the angle of attack, the yaw angle, and the immersion depth. The experimental results showed that both the torque and thrust coefficients increased with greater immersion depths and angles of attack. However, for the yaw angle, it was found that increasing the yaw angle led to an increase in the thrust coefficient but had minimal effect on the torque coefficient. Based on the optimization results, the optimum hydrodynamic coefficient and the maximum thrust coefficient of 0.0717 were determined, while the minimum torque coefficient is 0.0595. Based on this answer, the angle of attack, yaw angle, immersion depth and advance coefficient are determined as 3.4878, 5.1463, 0.4000 and 0.8890 respectively. An experimental test is performed in the proposed position. The results show that the optimization results and the experimental data have similar changes in the thrust and torque coefficients. This algorithm is well suited for the prediction of position and operating parameters, the advantage of this method can be mentioned leads to a reduction in the additional costs and time required for experimental tests. It is also suitable for problems with small amounts of data. The disadvantage of this method is that several models are examined in order to determine the most suitable ANN algorithm for generating new data. The future works that can be carried out are listed below:

- Investigation of various optimization methods such as Ant Colony Optimization (ACO), Particle Swarm Optimization (PSO),… can be examined and the results compared with this method
- This research can be tested with other intelligent methods such as Support Vector Machines (SVM) , Gaussian Processes (GP), … instead of ANN and compare the results with each other
- The experimental test can be performed with more position parameters to investigate their effects on the performance of the propeller

## Data Availability

The data that support the findings of this study are available from the corresponding author, [N.M. Nouri], upon reasonable request.

## Abbreviations

KT: Thrust coefficient
KQ: Torque coefficient
J: Advance coefficient
$${W}_{n}$$: Weber number
${\rho }_{v}$: Vapor pressure of water
$${\sigma }_{0}$$: Cavity number
$$F{r}_{n}$$: Froud number
$${I}_{T}$$: Immersion depth
D: Propeller diameter
$${Re}_{n}$$: Reynolds number
ψ: Yaw angle
α: Attack angle
AE/AO: Surface area ratio (expanded area/disk area)
η: Propeller efficiency
${Fr}_{n}$: Propeller Froude number
V: Advance velocity
Z: Number of blades
n: Rotation rate
ρ: Mass density of water
g: Gravitational constant
${h}_{T}$: Propeller immersion depth
ν: Kinematic viscosity
E: Elasticity module
T: Thrust
Q: Torque
P0: Static pressure on free surface water
